# Patient‐Derived Neurons Exhibit α‐Synuclein Pathology and Previously Unrecognized Major Histocompatibility Complex Class I Elevation in Mitochondrial Membrane Protein–Associated Neurodegeneration

**DOI:** 10.1002/mds.70029

**Published:** 2025-09-15

**Authors:** Leonie M. Heger, Leonie Kertess, Clara Kaufhold, Francesco Gubinelli, Aida Cardona‐Alberich, Gamze Özata, Stephan A. Müller, Sarah K. Tschirner, Oliver Stehling, Martina Schifferer, Camille Peron, Valeria Tiranti, Roland Lill, Arcangela Iuso, Luigi Zecca, Michael Strupp, Wolfgang Oertel, Stefan F. Lichtenthaler, Lena F. Burbulla

**Affiliations:** ^1^ Metabolic Biochemistry, Biomedical Center (BMC), Faculty of Medicine LMU Munich Munich Germany; ^2^ Neuroproteomics, School of Medicine and Health, Klinikum rechts der Isar Technical University of Munich Munich Germany; ^3^ German Center for Neurodegenerative Diseases (DZNE) Munich Germany; ^4^ Institute for Cytobiology Philipps University of Marburg Marburg Germany; ^5^ Centre for Synthetic Microbiology, Synmikro Marburg Germany; ^6^ Munich Cluster for Systems Neurology (SyNergy) Munich Germany; ^7^ Fondazione IRCCS Istituto Neurologico Carlo Besta Milan Italy; ^8^ Institute of Human Genetics, School of Medicine Technical University of Munich Munich Germany; ^9^ Institute of Neurogenomics Helmholtz Zentrum München Neuherberg Germany; ^10^ Institute of Biomedical Technologies National Research Council of Italy Segrate (Milan) Italy; ^11^ Department of Neurology LMU University Hospital, LMU Munich Munich Germany; ^12^ Department of Neurology Philipps University of Marburg Marburg Germany

**Keywords:** NBIA, MPAN, iPSC disease modeling, dopaminergic neurons, α‐synuclein

## Abstract

**Background:**

Mitochondrial membrane protein–associated neurodegeneration (MPAN) from the neurodegeneration with brain iron accumulation (NBIA) family is a rare neurodegenerative disease marked by α‐synuclein aggregation, brain iron accumulation, and midbrain dopaminergic neuron degeneration.

**Objective:**

The mechanisms driving neuron vulnerability remain unclear. Our study aimed to develop a patient‐derived disease model replicating key pathologies of patient brains.

**Methods:**

We generated induced pluripotent stem cell–derived midbrain dopaminergic neurons from MPAN patients and examined ultrastructural and biochemical markers of pathology.

**Results:**

MPAN patient neurons displayed α‐synuclein aggregation, axonal swellings, iron accumulation, and severe membrane destruction. In addition, levels of the major histocompatibility complex class I (MHC‐I), linked to cellular stress and neurodegenerative processes, were elevated in patient neurons. Treatment with acetyl‐leucine, a potentially neuroprotective compound, decreased MHC‐I.

**Conclusions:**

This first patient‐derived neuronal model of MPAN provides a useful tool for further research aimed at unraveling the complexities of this disease and developing potential therapeutic interventions. © 2025 The Author(s). *Movement Disorders* published by Wiley Periodicals LLC on behalf of International Parkinson and Movement Disorder Society.

Neurodegeneration with brain iron accumulation (NBIA) is a heterogenous group of neurological disorders characterized by progressive neurodegeneration and abnormal iron accumulation in the basal ganglia.^1^ About 5% to 10% of NBIA patients are affected by a subtype called mitochondrial membrane protein–associated neurodegeneration (MPAN) that shares clinical features with Parkinson's disease (PD),^2^, ^3^ including parkinsonism.^3^, ^4^ Postmortem brain analysis showed iron deposits predominantly in globus pallidus and substantia nigra (SN), degeneration of nigral dopaminergic neurons,^3^, ^5^ and axonal neuropathy.^3^, ^4^, ^5^ Further, MPAN patients experience pathological accumulation of α‐synuclein leading to Lewy body (LB) pathology—major hallmarks shared with PD, classifying both diseases as α‐synucleinopathies.^3^, ^5^, ^6^ MPAN patient brains even show greater density of LB pathology than those of patients with PD.^3^ For MPAN and PD, only symptomatic treatment is available to date.^7^

Mutations in *C19orf12* (chromosome 19 open reading frame 12), the causative gene in MPAN, lead to deficiency of a transmembrane protein associated with mitochondrial and endoplasmic reticulum membranes.^5^, ^8^ However, understanding of C19orf12's exact function is still limited. Some knowledge on the protein function has been acquired using simple human cell and animal models,^8^, ^9^, ^10^, ^11^, ^12^ or the first genetic MPAN mouse model (https://www.mousephenotype.org/data/genes/MGI:1919494). However, these models do not fully recapitulate human pathologies or explain mechanisms that cause midbrain neuron degeneration.

In this study, we examined midbrain‐specific dopaminergic (mDA) neurons generated from MPAN patient induced pluripotent stem cells (iPSCs) as human‐specific model system. These neurons exhibited key features of patient brain pathology—α‐synuclein and iron accumulation, axonal swellings, and membrane disruption. All of these are associated with increased expression of functional major histocompatibility complex class I (MHC‐I), which plays a key role in neuroinflammation associated with neurodegenerative diseases.^13^ MHC‐I levels were found to be elevated in MPAN patient neurons and, interestingly, decreased by acetyl‐leucine (AL), which was reported to have disease‐modifying effects in prodromal PD.^14^

Our findings provide insights into MPAN pathology and may facilitate the development of novel therapeutic approaches.

## Subjects and Methods

### Subjects

We used iPSCs from two female MPAN patients with homozygous *C19orf12* mutations (MPAN 1: FINCBi004‐A, MPAN 2: HMGUi004‐A)^15^ and two healthy female control subjects (control 1: STBCi053‐A, age of donor at collection: 64 years; control 2: STBCi052‐A, age of donor at collection: 57 years; hPSCreg). MPAN 1 showed disease onset at age 7 years, biopsy at 13 years, and death at 28 years. MPAN 2 showed onset at 7 years, biopsy at 22 years, and is currently 29 years old.

Informed consent was obtained from all participants. The study was conducted in accordance with the principles embodied in the Declaration of Helsinki and approved by the Institutional Review Boards of the Istituto Neurologico Carlo Besta, Italy, and the Ethics Committee of the Medical Faculty of LMU Munich (approval no. 25–0153), Germany.

Generation of iPSCs from patient fibroblasts was approved by the Ethics Committee of the Technical University of Munich (2022‐674‐S‐SR).

### Culture of Human iPSCs and Differentiation into Dopaminergic Neurons

iPSC cultures were maintained and differentiated into mDA neuronal cultures as previously described^16^, ^17^ and in the Supporting Information. Unless otherwise stated, mDA neuronal cultures at day 70 (d70) of differentiation were used.

### Biochemical Analysis of mDA Neuronal Cultures

Turnbull staining was performed according to published protocols (see Supporting Information).

For immunocytochemistry, cells were fixed in 4% formaldehyde for 15 minutes, permeabilized with 0.3% Triton X‐100, and blocked with 0.3% Triton X‐100/5% normal goat serum in PBS for 1 hour. Staining was performed using primary and secondary antibodies (see Supporting Information).

For live‐cell staining with tetramethylrhodamine ethyl ester (TMRE), MitoSOX Red, and MitoTracker Green, cells were seeded in 96‐well plates, stained, and imaged following manufacturers’ instructions (see Supporting Information).

Mitochondrial enzyme activities were analyzed in multiwell plates based on established assays as outlined in the Supporting Information.

Protein extraction and Western blot were performed using established protocols, as detailed in the Supporting Information.^17^ For treatment studies, neuronal cultures received 5 mM acetyl‐dl‐leucine (ADLL; #S451312; Sigma Aldrich), acetyl‐l‐leucine (ALL; #441511; Sigma Aldrich), or dimethylsulfoxide (DMSO) for 7 days (d63–d70 of differentiation).

Cultures for transmission (TEM) and scanning electron microscopy (SEM) were prepared according to the protocol (see Supporting Information). TEM micrographs were captured on a JEM 1400plus (JEOL) with an XF416 camera (TVIPS) and EM‐Menu software (TVIPS), and SEM micrographs on a DSM 950 (Zeiss).

### Statistics

Analysis of variance (ANOVA) with Tukey's post hoc test was performed; *P* values <0.05 were considered significant. Error bars represent standard error of the mean (SEM) (*P < 0.05, **P < 0.01, ***P < 0.001). Control 1 and control 2 values are combined as “CTRLs,” and MPAN 1, clones 1 and 2, are combined as “MPAN 1.” A minimum of three differentiations was used for statistical analysis. Single data points represent a biological replicate. In Figure 2D each data point represents an individual cell.

## Results

### α‐Synuclein Pathology and Iron Overload Phenotype in MPAN Patient Neurons Resemble Major Hallmarks of Human Brain Pathology

iPSCs of two MPAN patients carrying homozygous recessive mutations c.[139G>A], p.[Gly47Ser] (MPAN 1), and c.[161G>T], p.[Gly54Val] (MPAN 2) in C19orf12 (Supporting Information Fig. S1A‐B) and two controls (control 1, control 2) (Supporting Information Fig. S1B) were differentiated into mDA neurons (two clones, 1 and 2, for MPAN 1; one clone for all other lines) (Supporting Information Fig. S2A‐B).

**FIG. 1:**
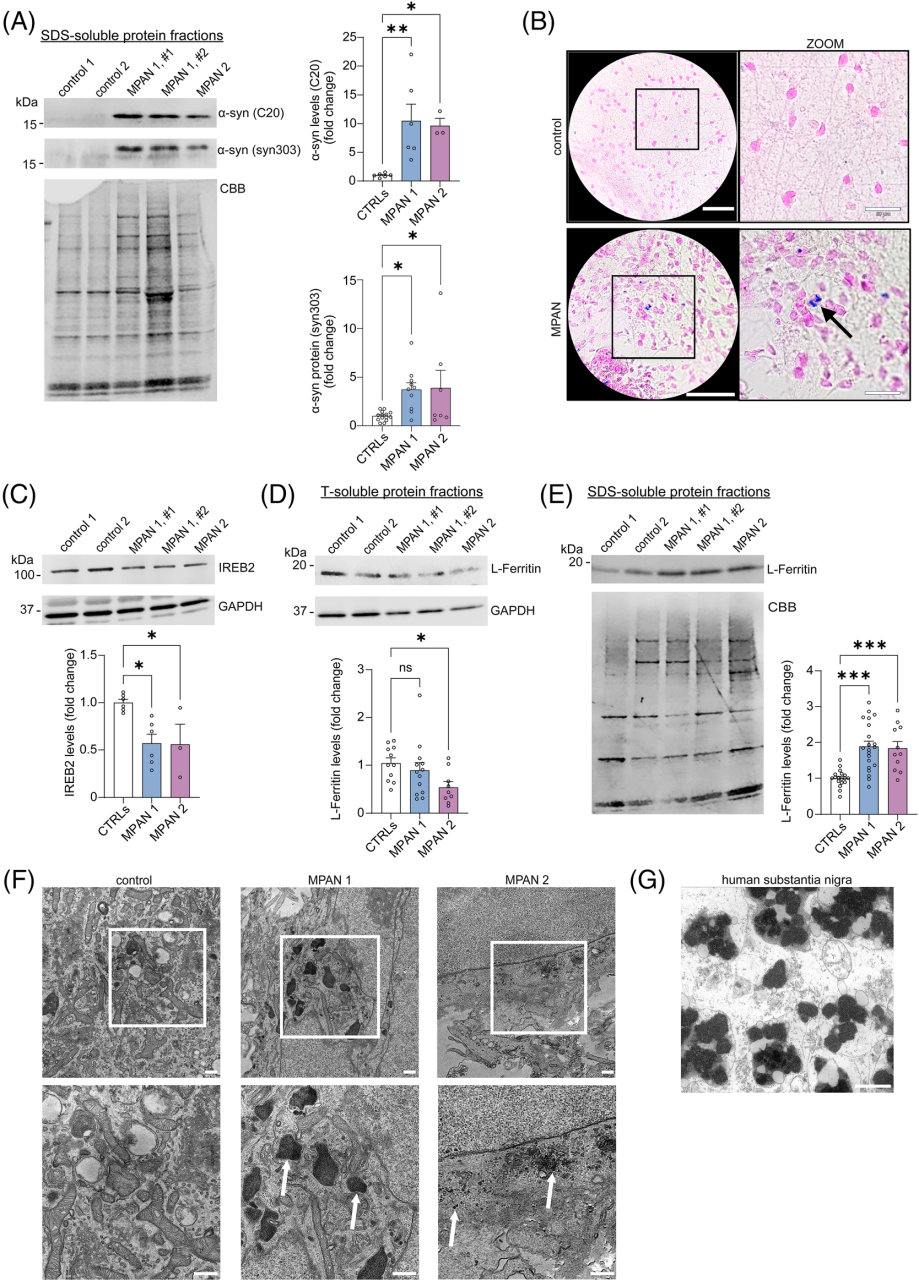
α‐Synuclein pathology and iron overload in membrane protein–associated neurodegeneration (MPAN) patient dopaminergic neurons. (**A**) Immunoblot analysis of all forms of α‐synuclein (C20 antibody) (n = 3–6) or oxidized/nitrated forms of α‐synuclein (syn303 antibody) (n = 7–14) in sodium dodecyl sulfate (SDS)‐soluble neuronal lysates from MPAN patient and control lines. Coomassie brilliant blue (CBB) was used as loading control. (**B**) Turnbull staining of control and MPAN patient neuronal cultures. Scale bars, 50 μm (low‐magnification images); 20 μm (high‐magnification/zoom images). Arrows indicate dark blue, iron‐dense granules. (**C** and **D**) Immunoblot analysis of (**C**) iron‐responsive element binding protein 2 (IREB2) (n = 3–6) and (**D**) L‐ferritin (n = 9–13) in Triton X‐100 (T)‐soluble neuronal lysates from MPAN patient and control lines. GAPDH was used as a loading control. (**E**) Immunoblot analysis of L‐ferritin in SDS‐soluble neuronal lysates of MPAN patient and control lines (n = 11–21). CBB was used as a loading control. (**F**) Representative transmission electron microscopy images depicting neuromelanin depositions (MPAN 1) (indicated by arrows) or neuromelanin precursor forms, likely iron–melanin–protein complexes (MPAN 2) (indicated by arrows), in patient neurons that were absent in control neurons. The boxed regions in the images on the top panel indicate the areas enlarged in the images on the bottom panel. Scale bars, 500 nm. (**G**) Electron microscopy image of dark neuromelanin organelles in human substantia nigra of an 89‐year‐old healthy subject. Scale bar, 1 μm. *P < 0.05, **P < 0.01, ***P < 0.001. α‐syn, α‐synuclein; CTRL, control; ns, not significant.

α‐Synuclein, a protein extensively studied in PD,^18^ but not MPAN, is known to accumulate into toxic species under pathological conditions.^19^, ^20^ Antibodies against all (C20) or preferentially pathological, disease‐associated (syn303) forms of α‐synuclein demonstrated sodium dodecyl sulfate (SDS)‐soluble α‐synuclein to be elevated in iPSC‐derived mDA neuronal cultures from both MPAN patients (Fig. 1A), whereas levels of Triton X‐100 (T)‐soluble α‐synuclein were solely elevated in MPAN 1 patient neuronal cultures (Supporting Information Fig. S3). In addition, truncated fragments of α‐synuclein, known to be pathophysiologically relevant, were observed in MPAN 1 patient mDA neuronal cultures (syn303, long exposure) (Supporting Information Fig. S3).^21^, ^22^

Next, we used Turnbull blue that positively stained iron aggregates in MPAN patient mDA neurons, but not control neuron cultures (Fig. 1B), a phenotype exacerbated upon iron treatment (Supporting Information Fig. S4A). Iron‐responsive element binding protein 2 (IREB2), degraded under excess iron, was diminished in patient neuronal cultures also indicating elevated iron concentration (Fig. 1C).

Increased cellular iron typically upregulates the iron‐binding protein ferritin. However, we detected decreased L‐ferritin levels in T‐soluble lysates of patient mDA neuronal cultures (Fig. 1D). The iron exporter ferroportin, normally upregulated under iron overload conditions, remained unchanged (Supporting Information Fig. S4B). Interestingly, L‐ferritin was elevated in SDS‐soluble fractions of MPAN patient mDA neuronal cultures (Fig. 1E), a phenotype closely linked to the formation of pathological iron deposits.^23^

Dopaminergic neurons in the human SN are characterized by high iron levels that catalyze the formation of the black‐brown polymer neuromelanin to sequester reactive metals and toxic dopamine metabolites, and reduce their toxicity.^24^, ^25^, ^26^ This process begins with the iron‐driven oxidation of cytosolic dopamine.^26^ Using TEM, we identified pigment‐dense structures in patient mDA neuronal cultures (Fig. 1F), resembling either enlarged autolysosomes or neuromelanin‐like organelles (MPAN 1), and smaller iron–melanin–protein complexes (MPAN 2). Notably, the organelles in MPAN 1 particularly resemble those found in human SN (Fig. 1G).

Following the hypothesis that mutations in *C19orf12* appear to be associated with mitochondrial defects and that dysbalanced iron may exacerbate those, we assessed the mitochondrial status. However, mitochondrial membrane potential (TMRE staining), mitochondrial oxidative stress (mitoSOX staining), and the activity of the mitochondrial iron–sulfur and heme enzymes were not significantly changed between patient and control neuronal cultures (Supporting Information Fig. S5A,B). Although mitochondrial defects may not be the primary pathology driver in MPAN, α‐synuclein and iron accumulation may affect proper cellular function.

### Axonal Swellings and Elevated MHC‐I Levels in MPAN Patient Neurons

**FIG. 2:**
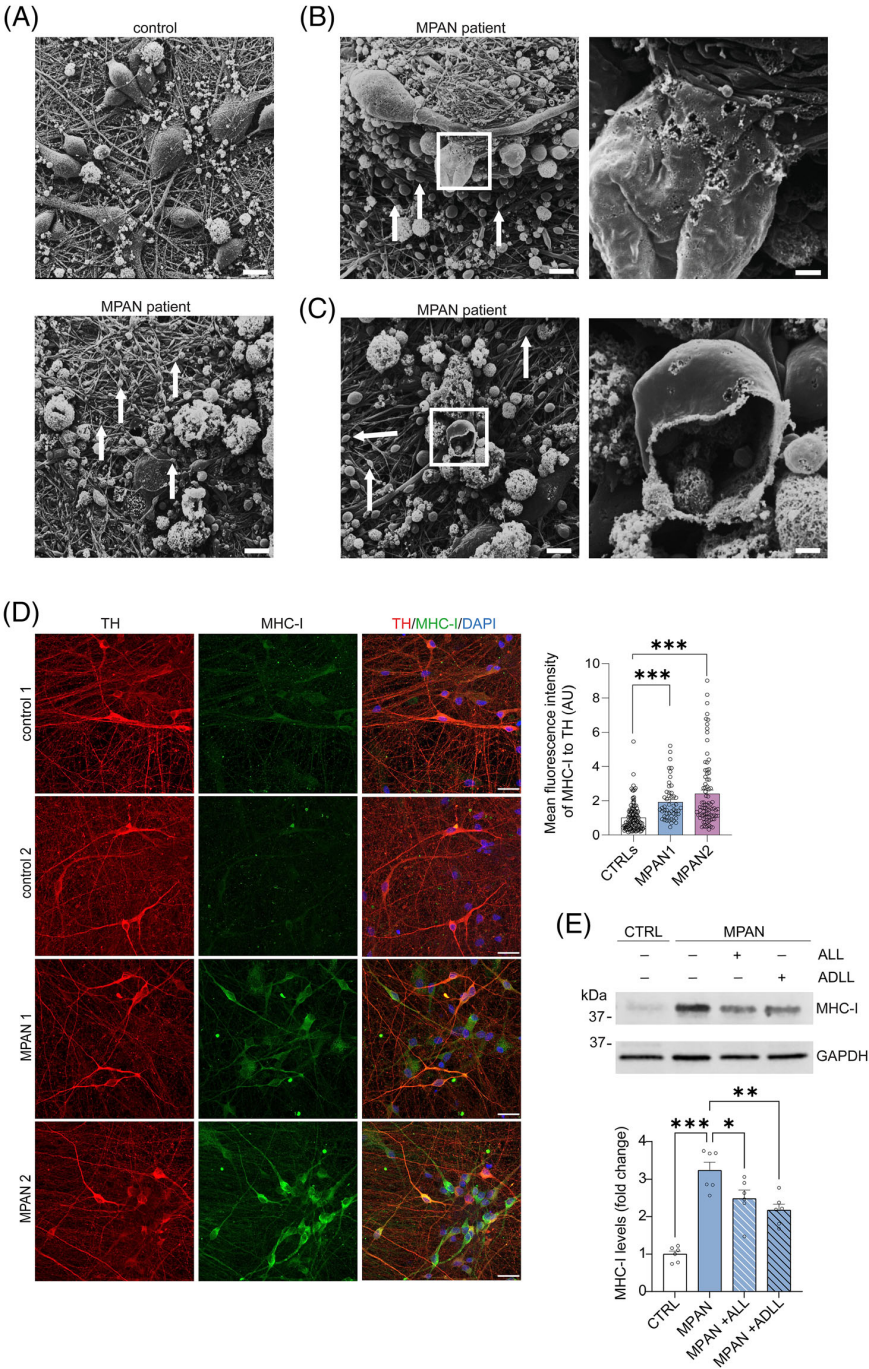
Axon pathology and elevated major histocompatibility complex class I (MHC‐I) levels in dopaminergic neurons from membrane protein–associated neurodegeneration (MPAN) patients, with acetyl‐leucine (AL) reducing MHC‐I. (**A**) Representative scanning electron microscopy image of MPAN patient and control dopaminergic neuronal cultures depicting numerous axonal swellings in MPAN patient neurons (indicated by arrows) that were absent in control neurons. Scale bars, 10 μm. (**B** and **C**) Representative scanning electron microscopy images of MPAN patient neuronal cultures with (**B**) moderately disrupted cellular membranes (indicated in zoom) or (**C**) complete disruption of cellular membranes. Axonal swellings are indicated by arrows. The boxed regions in the images on the left indicate the areas enlarged in the images on the right. Scale bar, 5 μm (low‐magnification images); 1 μm (high‐magnification/zoom images). (**D**) Immunostaining and quantification of MHC‐I fluorescence intensity (green) in tyrosine hydroxylase (TH)‐expressing (red) control or MPAN patient dopaminergic neuronal cultures. Merged staining shows DAPI as nuclear stain. Scale bars, 20 μm (n = 51–120 cells/cell line). (**E**) Immunoblot analysis of MHC‐I in Triton X‐100 (T)‐soluble neuronal lysates from control, MPAN patient, and MPAN patient treated for 7 days with 5 mM of either acetyl‐l‐leucine (ALL), acetyl‐dl‐leucine (ADLL), or carrier dimethylsulfoxide (DMSO) (n = 6). GAPDH was used as loading control. *P < 0.05, **P < 0.01, ***P < 0.001. CTRL, control.

Toxic insults such as dysregulated α‐synuclein or iron mishandling cause axonal damage and degeneration.^27^ Focal axon swellings and larger spheroids are early pathological findings in PD^28^ and MPAN,^3^, ^5^ and most evident in regions with high α‐synuclein load.^29^ SEM showed axonal swellings in MPAN patient mDA neuronal cultures (Fig. 2A), often correlated with disrupted (Fig. 2B; Supporting Information Fig. S6) or completely destructed membranes (Fig. 2C).

Notably, protein accumulation, axonal pathology, and other neurotoxic insults are triggers of MHC‐I expression, which is known to play a role in neurodegenerative diseases by contributing to neuroinflammation.^13^ In fact, we detected elevated MHC‐I levels in tyrosine hydroxylase–positive mDA neuronal cultures from MPAN patients via immunocytochemical (Fig. 2D) and immunoblotting analyses (Fig. 2E).

Next, we used the modified amino acid AL, specifically the racemate acetyl‐DL‐leucine (ADLL) and the bioactive enantiomer acetyl‐L‐leucine (ALL), to modulate this pathological feature. Both ADLL and ALL reduced MHC‐I levels in patient mDA neuronal cultures (Fig. 2E), offering a strategy for reducing pathological triggers of disease.

## Discussion

Although MPAN shares certain pathological features with PD, it remains a genetically and clinically distinct condition. Modeling MPAN is therefore essential to uncover disease‐specific mechanisms of this rare neurodegenerative disorder. In this study, we report the first patient‐derived neuronal model of MPAN that, importantly, recapitulates typical features of brain pathology, i.e., elevated α‐synuclein, iron deposits, and axonal pathology.

It has long been discussed whether brain iron accumulation in MPAN, and other NBIA subtypes, is a cause or consequence of the disease. Although our findings indicate increased intracellular iron in patient mDA neuronal cultures, this overload cannot be explained by canonical iron regulatory system alterations. Previous findings relate to the role of metal dysregulation in the development and progression of neurodegenerative diseases, such as PD.^23^, ^30^ The presence of reactive iron may contribute to cellular damage, including membrane disruption via Fenton reaction‐induced lipid peroxidation,^31^ a possible cause of membrane damage in MPAN patient neuronal cultures. Additional studies are needed to elucidate the molecular pathways behind the observed iron anomalies.

In the mature human brain, neuronal MHC‐I expression is minimal except in catecholaminergic neurons of the SN and locus coeruleus, where it can be induced by specific stimuli.^32^ Interestingly, catecholaminergic neurons, including dopaminergic neurons, are particularly susceptible to MHC‐I induction.^32^ Neurotoxin models of PD show that oxidative stress triggers MHC‐I expression and cytotoxic T‐cell infiltration.^33^ Although neuroinflammation is implicated in other NBIA subtypes with dopaminergic neuron loss,^34^, ^35^ its role in MPAN remains unexplored. Further studies are needed to identify MHC‐I triggers and assess inflammatory contributions to neuron vulnerability in MPAN.

Nevertheless, we used elevated MHC‐I levels as an indicator of intracellular stress in MPAN patient mDA neuronal cultures to guide our rescue strategy with AL. Our results indicate that AL treatment successfully lowered MHC‐I levels, suggesting mitigation of stress‐related cellular triggers. Just recently, AL was suggested to have disease‐modifying properties in prodromal PD.^14^ It also improves symptoms and pathology in models of traumatic brain injury and rare lysosomal disorders.^36^, ^37^, ^38^, ^39^ Although the mechanism of action discussed for AL is to correct metabolic and lysosomal dysfunction,^40^ the exact mechanism for the observed MHC‐I reduction in our study necessitates further in‐depth analysis.

Patient‐derived neuronal models provide a critical platform to unravel the complexities of MPAN and explore therapeutic strategies. Our work represents a crucial step toward enabling such advanced research.

## Author Roles

Author roles: (1) Conception and Design of the Study, (2) Acquisition and Analysis of Data, (3) Drafting a Significant Portion of the Manuscript or Figures, (4) Reviewing and Editing the Manuscript. L.M.H.: 1, 2, 3 L.K.: 1, 2, 4 C.K.: 2, 3, 4 F.G.: 2, 4 A. C.‐A.: 2, 4 G.Ö.: 2, 3, 4 O.S.: 2, 4 M. Schifferer: 2, 4 C.P.: 1 V.T.: 1, 4 A.I.: 1, 4 R.L.: 1, 4 L.Z.: 1, 4 M. Strupp: 1, 4 W.O.: 1, 4 S.A.M.: 1, 2, 4 S.K.T.: 1, 2 S.F.L.: 1, 4 L.F.B.: 1, 3, 4

## Full Financial Disclosures for the Previous 12 Months

L.M.H., L.K., C.K., F.G., A. C.‐A., G.Ö., S.A.M., S.K.T., O.S., C.P., V.T. and S.F.L. had no specific financial support that relates to the research discussed in the submitted article and no other funding source in the previous 12 months and thus no potential conflicts of interest. M. Schifferer has received funding by the Deutsche Forschungsgemeinschaft (DFG, German Research Foundation), TRR 274/2, 408885537 (project Z01). A.I. has received funding by the NBIA Disorders Association, Hoffnungsbaum e.V., NBIA Suisse, and the Bavarian Research Alliance (BayIntAn_TUM_MRI_2025_03) L.Z. has received funding by the Pezzoli Foundation for Parkinson’s disease‐Milano and PANSIMS project C21/BM/15754743 by Luxembourg National Research Fund. R.L. has received funding by the Deutsche Forschungsgemeinschaft (DFG, German Research Foundation). W.O. has received speaker’s honoria on educational symposia sponsored by Abbvie, the International Movement Disorders Society and Stada Pharma. He is a member of advisory boards with the companies Intrabio and MODAG and holds stock options with Intrabio. The institution of W.H.O., not W.H.O personally received/s scientific grants from ParkinsonFonds, Deutschland/ Stichting ParkinsonFonds, The Netherlands, from the German Research Foundation and the Michael J Fox Foundation ‐ all unrelated to the manuscript. M. Strupp received support for clinical studies from Decibel, U.S.A., Cure within Reach, U.S.A. and Heel, Germany. He acts as a consultant for Abbott, AurisMedical, Bulbitec, Heel, Sensorion, Vifor and Vertify. He is a scientific founder, investor and share‐holder of IntraBio. L.F.B. has received funding from the European Research Council (ERC) under the European Union’s Horizon 2020 research and innovation programme (grant agreement No. [948027]), from the Deutsche Forschungsgemeinschaft (DFG, German Research Foundation) under the Heisenberg Programme (Project No. 447395247), and from the Rise up! programme of the Boehringer Ingelheim Stiftung (BIS).

## Supporting information


**Data S1.** Supporting Information.

## Data Availability

The data underlying this article are available in the article and in its Supporting Information.
