# Supplementary material for: Patient‐Derived Neurons Exhibit α‐Synuclein Pathology and Previously Unrecognized Major Histocompatibility Complex Class I Elevation in Mitochondrial Membrane Protein–Associated Neurodegeneration
Source: Mov Disord. 2025 Sep 15;40(12):2811–8. doi: 10.1002/mds.70029 (PMC12710160; doi:10.1002/mds.70029)
Supplement: Supplementary file 1 — Data S1. Supporting Information. [file MDS-40-2811-s001.docx]

**SUPPLEMENTAL INFORMATION for**

**Title**: Patient‐Derived Neurons Exhibit α‐Synuclein Pathology and Previously Unrecognized Major Histocompatibility Complex Class I Elevation in Mitochondrial Membrane Protein–Associated Neurodegeneration

**Authors:** Leonie M. Heger, MSc,^1*^ Leonie Kertess, PhD,^1*^ Clara Kaufhold, DVM,^1^ Francesco Gubinelli, PhD, ^1^ Aida Cardona-Alberich, PhD,^1^ Gamze Özata, MSc,^1^ Stephan A. Müller, PhD,^2,3^ Sarah K. Tschirner, PhD,^2,3^ Oliver Stehling, PhD,^4,5^ Martina Schifferer, PhD,^3,6^ Camille Peron, MSc,^7^ Valeria Tiranti, PhD,^7^ Roland Lill, PhD,^4,5^ Arcangela Iuso, PhD,^8,9^ Luigi Zecca, MD,^10^ Michael Strupp, MD,^11^ Wolfgang Oertel, MD,^9,12^ Stefan F. Lichtenthaler, PhD,^2,3,6^ Lena F. Burbulla, PhD^1,3,6#^

**Correspondence to: Lena.Burbulla@med.uni-muenchen.de**

**This file includes:**

Supplementary Figures S1-S6

Supplemental Material and Methods

**
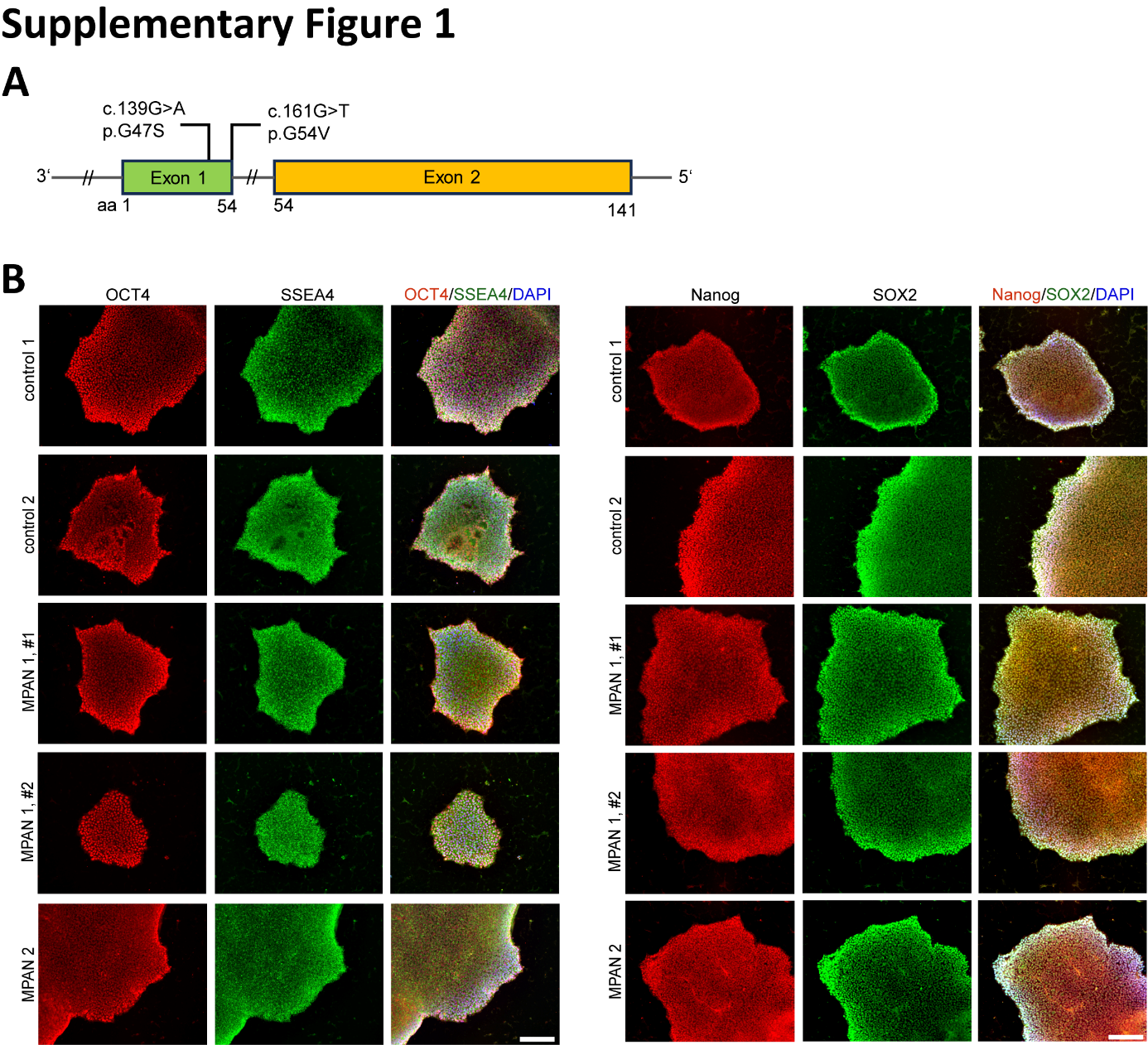
**

**Supplementary figure 1: Characterization of iPSC lines from MPAN patients and controls.** (**A**) Structure of the *C19orf12* gene and schematic overview of sites of mutation c.[139G > A], p.[Gly47Ser] in cells from patient MPAN 1 and c.[161G > T], p.[Gly54Val] in cells from patient MPAN2. (**B**) Immunocytochemistry for pluripotency markers OCT4, SSEA4, NANOG and SOX2 and nuclear counterstaining with DAPI in iPSCs from two healthy individuals (control 1, control 2), from patient MPAN 1 (two clones, #1 and #2) and from patient MPAN 2. Scale bar, 180μm.


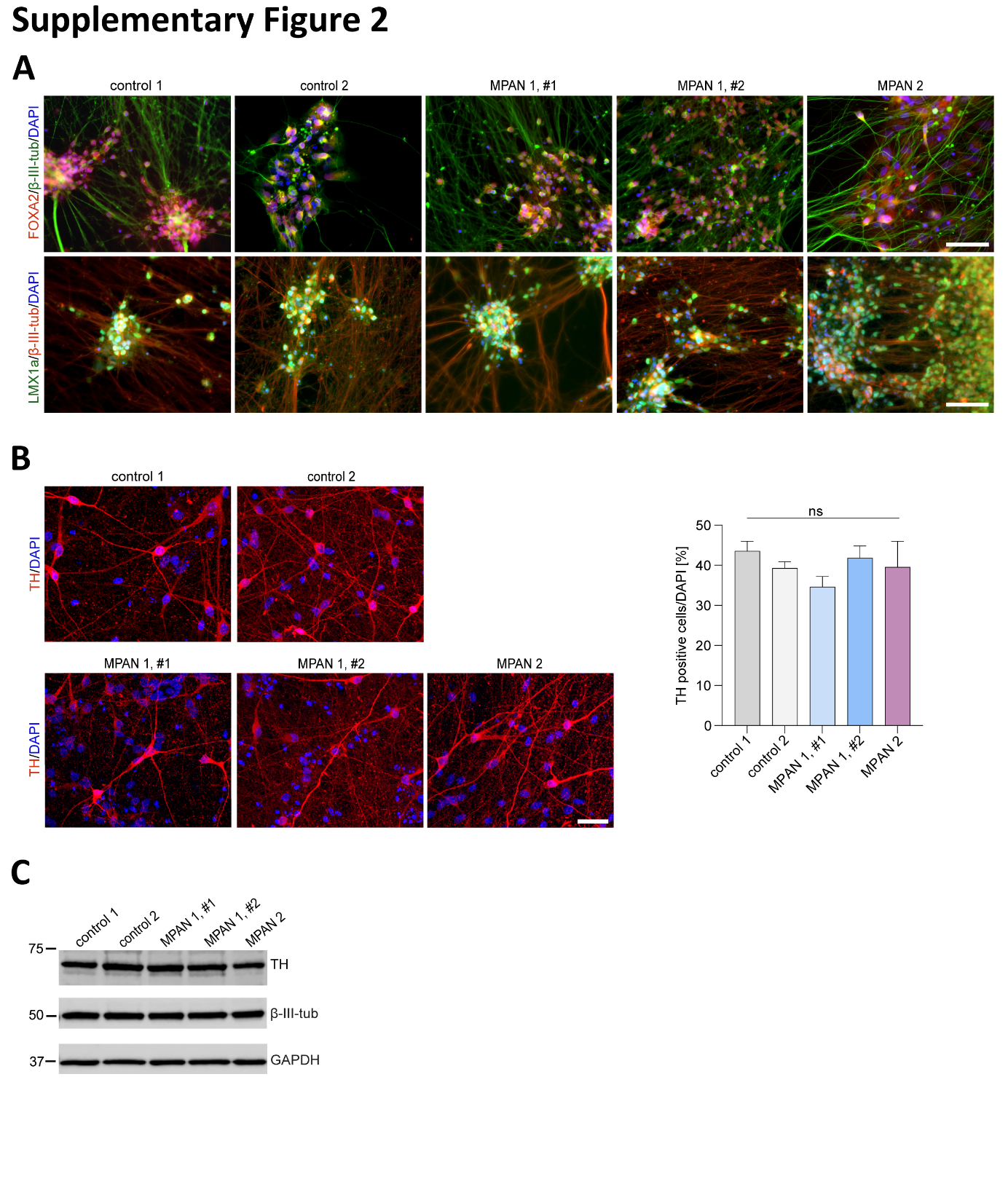


**Supplementary figure 2: Characterization of differentiated dopaminergic neurons from MPAN patients and controls.** (**A**) Immunocytochemistry of midbrain and neuronal markers in iPSC-derived dopaminergic neurons from MPAN patient and control lines at day 30 (d30) of differentiation. Antibodies against FOXA2, LMX1a and β-III-tubulin were used; nuclei were counterstained with DAPI. Scale bar, 50μm. (**B**) Immunocytochemistry and quantification of the percentage of TH-positive cells in iPSC-derived dopaminergic neuron cultures from MPAN patient and control lines at d70 of differentiation. Antibody against TH was used; nuclei were counterstained with DAPI. Scale bar 30μm. (**C**) Immunoblot analysis of TH and β-III-tubulin levels in T-soluble neuronal lysates from MPAN patient neurons as well as control neurons at d30 of differentiation. GAPDH was used as a loading control.

**
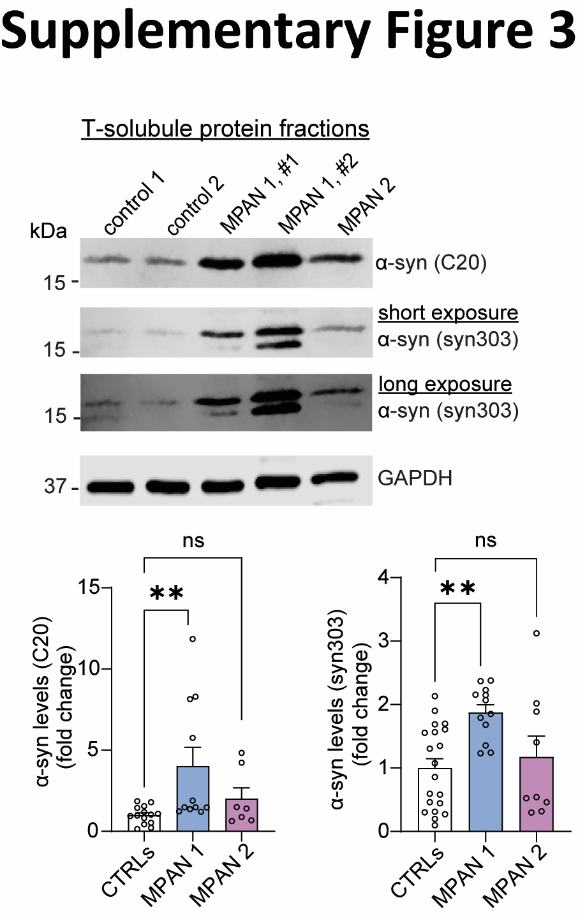
**

**Supplementary figure 3: Levels of T-soluble α-synuclein in immunoblotting studies.** Immunoblot analysis of all forms of α-synuclein (C20 antibody) (n = 7-14) or oxidized/nitrated forms of α-synuclein (syn303 antibody) (n = 9-20) in T-soluble neuronal lysates from MPAN patient and control lines, and quantification. GAPDH was used as loading control.

**
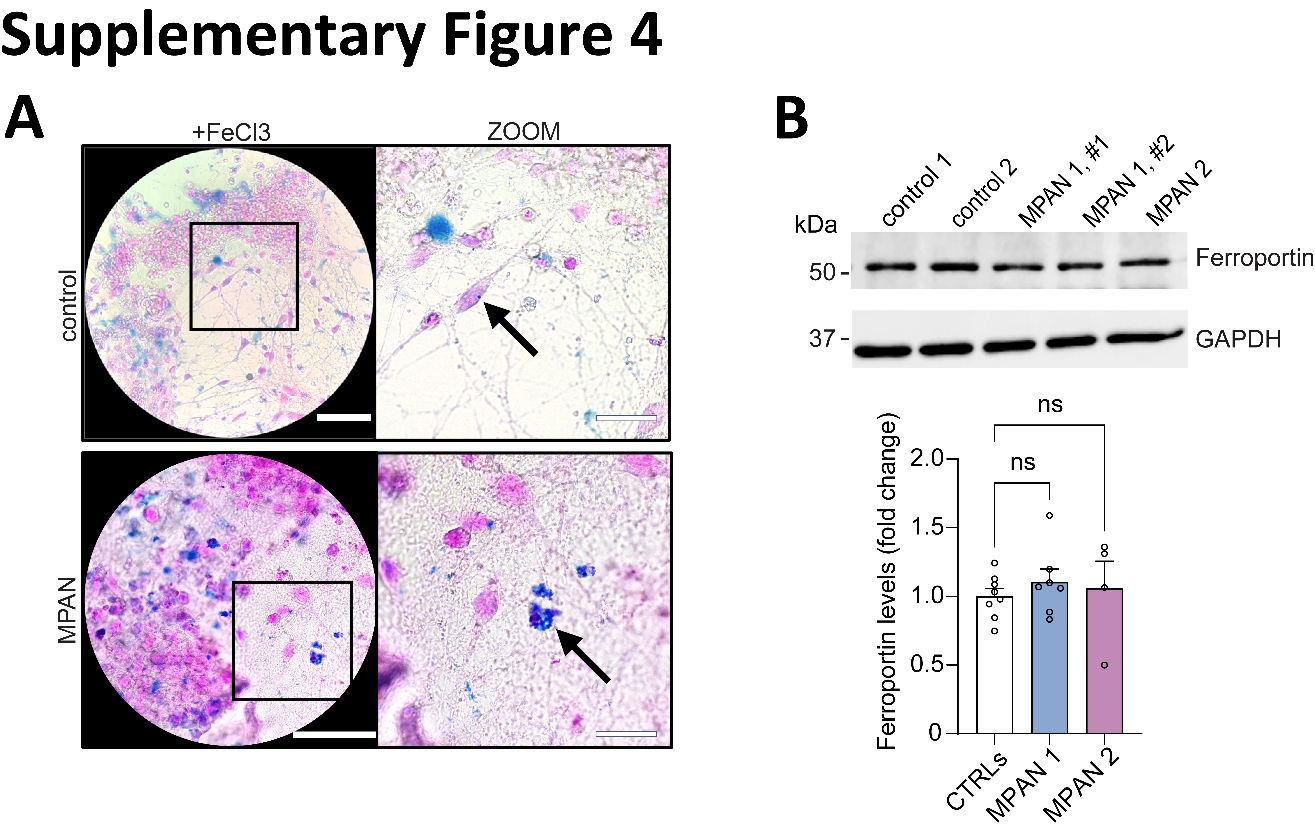
**

**Supplementary figure 4: Iron phenotype in MPAN patient neurons**. (**A**) Turnbull staining of control and MPAN patient neurons treated with FeCl_3_. Scale bar, 50µm (low magnification images), Scale bar, 20µm (high magnification/zoom images). Arrows indicate dark blue, iron-dense granules. (**B**) Immunoblot analysis of ferroportin in T-soluble neuronal lysates of control and MPAN patient lines, and quantification (n = 4-8). GAPDH was used as loading control.

**
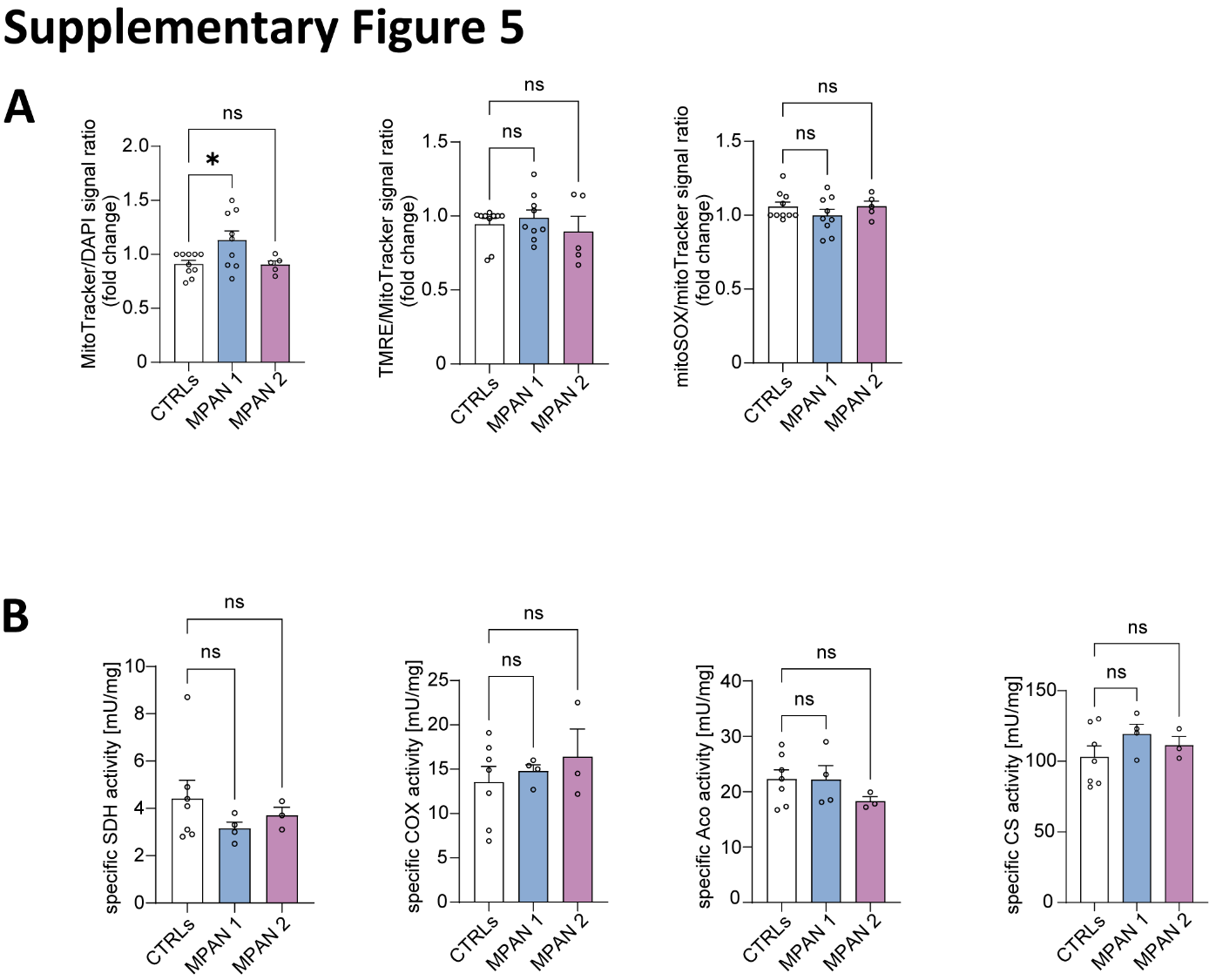
Supplementary figure 5: Analysis of mitochondrial status and mitochondrial iron-sulfur enzymes.** (**A**) (left) Measurement of MitoTracker fluorescence signal of MPAN patient and control neurons. The results were normalized to DAPI (n = 5-10). (middle) Measurement of TMRE fluorescence signal of MPAN patient and control neurons. TMRE fluorescence correlates with mitochondrial membrane potential. The results were normalized to MitoTracker signal (n = 5-10). (right) Measurement of mitoSOX red fluorescence signal. Fluorescence signal correlates with amount of mitochondrial reactive oxygen species. The results were normalized to MitoTracker signal (n = 5-10). (**B**) Total cell lysates of MPAN patient and control neurons were analyzed for the specific enzyme activities of succinate dehydrogenase (SDH, respiratory complex II), cytochrome c oxidase (COX, respiratory complex IV), aconitase (Aco), and citrate synthase (CS), as indicated (n = 3-7).

**
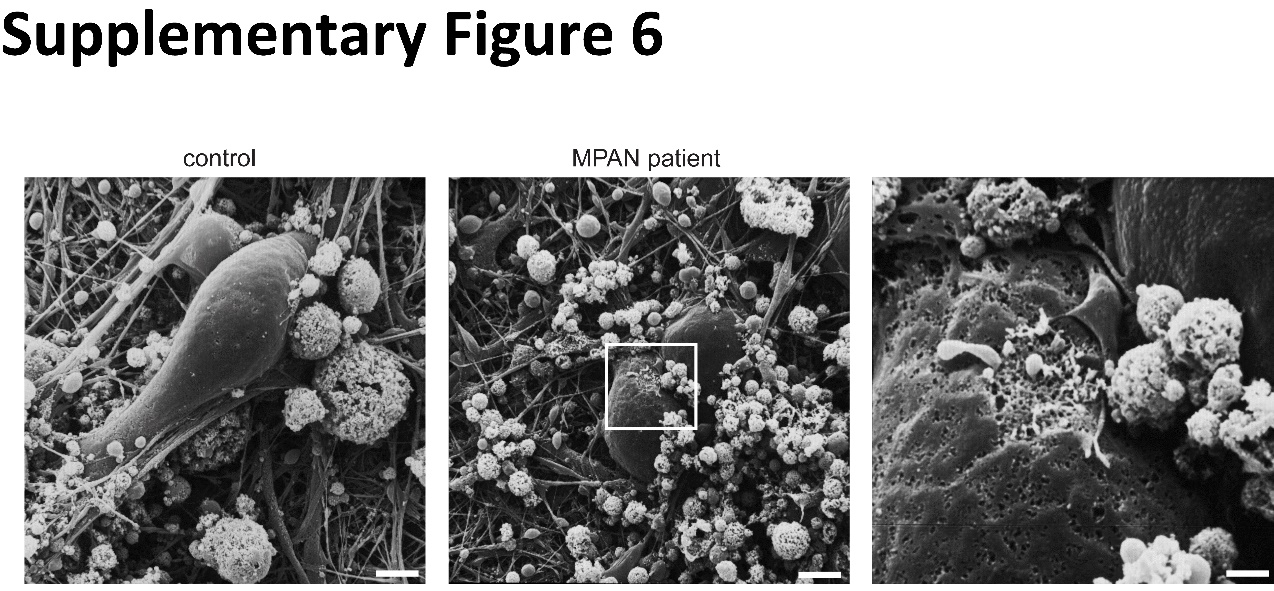
**

**Supplementary figure 6: Membrane disruptions in MPAN patient dopaminergic neurons.** Representative scanning electron microscopy images of control and MPAN patient dopaminergic neurons depicting membrane disruptions in MPAN patient neurons (indicated in zoom) that were absent in control neurons. The boxed region in the image in the middle indicate the area enlarged in the image on the right. Scale bar, 5µm (low magnification image), Scale bar, 1µm (high magnification/zoom images).

**Material and Methods**

**Culture of human iPSCs and differentiation into dopaminergic neurons**

iPSC cultures were maintained on Cultrex-coated (R&D Systems) plates in antibiotic-free mTESR Plus medium (Stem Cell Technologies). Cells were manually passaged every 5-7 days. If required, colonies were first manually groomed under a stereomicroscope to remove regions of spontaneous differentiation using a fine-tipped pipette prior to passaging. Colonies were then manually fragmented by scoring a crosshatch pattern across the colony surface with a P200 pipette tip to generate smaller aggregates. Subsequently, colony fragments were gently dislodged and transferred to newly prepared Cultrex-coated wells with fresh culture medium at a density of 1–2 colonies per well to enable continued propagation. Wells were gently swirled to distribute fragments evenly before incubation at 37°C in a humidified atmosphere containing 5% CO₂. After 48h, the culture medium was replaced with fresh mTeSR™ Plus and subsequently changed every 48h. All lines were routinely tested for mycoplasma contamination.

For differentiation into midbrain dopaminergic neurons, iPSCs were cultured mTESR Plus medium until >95% confluence (Day 0 = d0). To initiate differentiation, media was changed to Knockout DMEM containing 15% Knockout Serum Replacement (KOSR), 1X penicillin-streptomycin, 1X L-glutamine, and 1mM beta-mercaptoethanol (KSR media) supplemented with LDN193189 (LDN) and SB431542 (SB). On day 1, recombinant human sonic hedgehog (SHH), purmorphamine (PUR) and recombinant human FGF-8a (FGF8) were added to the media, and on day 3 stemolecule CHIR99021 (CHIR) was further added the media composition. On day 5, media was switched NbSm1 media (Neurobasal media, Neurocult SM1, 1X penicillin-streptomycin, and 1X L-glutamine, supplemented with LND, SHH, PUR, FGF8 and CHIR). On day 7, SHH, PUR and FGF8 were removed from the media. Between days 11-14, cells were passaged as 1-2mm chunks onto poly-d-lysine (PDL)/laminin-coated dishes in NbSm1 media supplemented with CHIR, recombinant human BDNF (B), ascorbic acid (A), recombinant human GDNF (G), recombinant human TGF-b3 (T), dibutyryl-cAMP (C), and DAPT (D). Between days 25-30, cells were rendered to single cell suspension with accutase, and passaged onto PDL/laminin-coated dishes for final experiments in NbSm1 media supplemented with B, A, G, T, C, D, and maintained in this media composition until days 40-50, after which neurons were cultured in NbSm1 alone until collection day.

**Turnbull staining**

We applied a modified Turnbull stain to iPSC-derived neuron cell cultures according to the manufacturer’s protocol. Cells were fixed in 4% formaldehyde for 15 min and stored in 1% PBS solution until further processed with Turnbull Blue stain after Quincke reaction. For iron overload condition, neurons were treated for 24h with 100μM FeCl_3_ (Merck, 701122) solution.

**Immunofluorescent staining and image analysis**

Glass coverslips were coated with poly-D-lysine (PDL) and laminin prior to plating neurons at a density of 3 × 10⁵ cells per well in a 24-well plate. On d70 of differentiation, cultures were washed once with ice-cold PBS and fixed with 4% paraformaldehyde (PFA) for 15–20min at room temperature. Fixed cells were washed three times with PBS and stored at 4°C in PBS if not processed immediately. Permeabilization was carried out using PBS containing 0.3% Triton X-100 (PBS-T) for 10min, followed by blocking with 5% normal goat serum and 1% defatted BSA in PBS-T for 1h at room temperature. Primary antibodies including Oct4 (Abcam #ab19857, 1:200), SOX2 (Abcam #ab79351, 1:200), Nanog (Abcam #ab80892, 1:200), SSEA-4 (Millipore #MAB4304 at 1:200), TuJ (ß-III-Tubulin) (Biolegend #801202, 1:1000; Biolegend #802001, 1:1000), LMX1a (Abcam #ab10533; 1:1000), FOXA2 (Santa Cruz #sc-101060; 1:200), TH (Tyrosine Hydroxylase) (Millipore #AB152; 1:500) and anti-HLA Class 1 ABC (Abcam #ab70328; 1:100) (abcam #138501; 1:200) were diluted in blocking buffer and left in a humidified chamber overnight at 4°C. After three PBS washes, secondary antibodies conjugated to Alexa Fluor® 488 or 568 (Invitrogen #A11036; 1:500; Invitrogen #A11029; 1:500) were applied for 1h at room temperature. Coverslips were washed twice with PBS and once with water before being mounted onto slides using DAPI-containing mounting medium. Mounted samples were dried overnight at 4°C protected from light prior to imaging.

Pictures were taken with brightfield and fluorescence microscope (ECHO Revolve, Discover Echo, San Diego, CA, USA) or confocal microscope (Leica TCS SP5, Leica Microsystems, Wetzlar, Germany). For all ICC analysis, at least three fields of view were analyzed per coverslip. For HLA Class I ABC (MHC-I) staining, in each field of view, the somas of neurons positively stained for TH (3 to 9 TH-positive cells per field) were marked, and the HLA fluorescence intensity of each individual cell was quantified using the Corrected Total Cell Fluorescence (CTCF) method.

**MitoSOX Red, TMRE and MitoTracker Green live-cell staining**

For live-cell staining, 100,000 cells per well were seeded on days 25–30 in 96-well plates (Falcon® 96-well Black/Clear Flat Bottom TC-treated Imaging Microplate) and maintained in culture until days 80–90. Staining procedures followed the manufacturers' instructions unless otherwise specified.

MitoSOX Red (Invitrogen, #M36008) was used at 1 μM, TMRE (Abcam, #ab113852) was diluted to 200 nM, MitoTracker Green (Invitrogen, #M7514) was used at 200 nM. Cell nuclei were stained with 2 drops/mL Hoechst 33342 (Invitrogen, #R37605). Dilutions were prepared in culture medium, with 100 μL/well added and incubated for 30min.

Photometric analysis followed two PBS or 0.2% BSA in PBS washes, using excitation/emission wavelengths of 396–15/610–20 nm (MitoSOX Red), 540–15/584–20 nm (TMRE), 481–15/525–20 nm (MitoTracker Green), and 360–20/460–30 nm (Hoechst 33342).

**Activity assays of mitochondrial enzymes**

Cultured neurons were harvested by scraping in cold PBS, centrifuged at 500x g for 10min and snap-frozen until use. For enzyme activity measurements cell pellets were resuspended in a digitonin-containing isosmotic sucrose buffer to support membrane permeabilization^2^, and protein concentration was determined by the BCA method. Enzyme activities were analyzed in multi-well plates based on established assays.^3,4^ Briefly, SDH activity was determined by a dichlorophenol-indophenol reduction assay, COX activity by oxidation of cytochrome-c, aconitase activity by coupling to isocitrate dehydrogenase-dependent reduction of NADP+, and CS activity by use of Ellmann’s reagent.

**Protein extraction and western blot analysis**

Cultured neurons were harvested by scraping in cold PBS and centrifuged at 500x g for 5min. Cell pellets were sequentially homogenized using 1% Triton™ X-100 lysis buffer (containing 10% glycerol, 150 mM NaCl, 25 mM HEPES pH 7.4, 1 mM EDTA, 1.5 mM MgCl2, proteinase inhibitor cocktail), to isolate the “T-soluble” fraction, followed by further extraction with 2% SDS/50mM Tris pH 7.4 by boiling and sonication to isolate the “SDS-soluble fraction”. Purified proteins were quantified by bicinchoninic acid (BCA) assay, then 10ug were run via SDS-PAGE on gradient gels under reducing conditions. Gels were blotted onto polyvinylidene difluoride (PVDF) membranes and blocked with Licor Intercept blocking reagent. Primary antibodies used: anti-GAPDH (1:10000; Millipore #MAB374), anti-TUJ1 (1:10000; BioLegend #802001; 801201), anti-tyrosine hydroxylase (1:1000; Santa Cruz #sc-25269, Millipore #AB152), anti-L-Ferritin (1:200; Santa Cruz #sc-74513), anti-IREB2 (1:500; ProteinTech #29986-1-AP), anti-ferroportin (1:1000; Invitrogen #PA5-22993), anti-alpha-synuclein (Syn303; 1:1000; BioLegend #824301; C-20; 1:1000; Santa Cruz #sc-7011-R), anti-HLA Class 1 ABC (1:1000; Abcam #ab70328). Blots were then probed with the appropriate fluorescent secondary antibody (IRDye, Li-Cor) and imaged with the Odyssey Fc Imaging System (Li-Cor).

**Transmission electron microscopy (TEM)**

For TEM of neuronal cells, we positioned freshly plasma-coated ACLAR® (plastic) films (Science Services) into the cell culture dish before seeding. 5% glutaraldehyde (EM-grade, Science Services) in 0.2M cacodylate buffer pH 7.4 (Science Services) prewarmed to 37°C was added 1:1 to cell culture medium and replaced by 2.5% glutaraldehyde in 0.1M cacodylate buffer after 5 min. Dishes were incubated for further 25 min on ice. Cells were washed 3x 5 min with 0.1 M cacodylate buffer on ice and stored in buffer at 4°C until postfixation in reduced osmium (1% osmium tetroxide (Science Services), 0.8% potassium ferrocyanide (Sigma Aldrich) in 0.1 M sodium cacodylate buffer). After contrasting in aqueous 0.5% uranylacetate (Science Services), the cells were dehydrated in an ascending ethanol series, infiltrated in epoxy-araldite resin LX112 (Ladd Research) and cured for 48h at 60 °C. Ultrathin sections were deposited onto formvar-coated copper grids (Plano) without postcontrasting. TEM micrographs were acquired on a JEM 1400plus (JEOL) equipped with a XF416 camera (TVIPS) and the EM-Menu software (TVIPS). Image analysis was performed in Fiji^5^. TEM image of neuromelanin organelles in human substantia nigra was derived from an 89-years-old male healthy subject. For tissue treatment and ethic policies see Zucca et al.^6^

**Scanning electron microscopy (SEM)**

Patient and control neuron samples were initially fixed in a 1,5% glutaraldehyde solution, followed by immersion in 1% osmium tetroxide, as [per Caulfield (1957)](https://rupress.org/jcb/article-pdf/3/5/827/1618333/827.pdf) (Ibid., 3 (1957), p. 827). Dehydration was achieved through a progressive series of acetone baths. Subsequently, critical point drying was performed using carbon dioxide (Balzers Union Point Dryer CPD 030, Lichtenstein). The specimens were then prepared on aluminum stub holders (Plano, Marburg) coated with conductive tabs (Plano, Marburg) and sputter-coated with an approximately 12 nm thick layer of gold/palladium alloy (Balzers Union SCD 040, Lichtenstein). Evaluation was carried out using a scanning electron microscope (DSM 950, Zeiss Oberkochen).

**Materials and Methods References**

1 Owen, J. E., Bishop, G. M. & Robinson, S. R. Uptake and Toxicity of Hemin and Iron in Cultured Mouse Astrocytes. *Neurochem Res* **41**, 298-306, doi:10.1007/s11064-015-1795-7 (2016).

2 Stehling, O., Paul, V. D., Bergmann, J., Basu, S. & Lill, R. Biochemical Analyses of Human Iron-Sulfur Protein Biogenesis and of Related Diseases. *Methods Enzymol* **599**, 227-263, doi:10.1016/bs.mie.2017.11.004 (2018).

3 Stehling, O. *et al.* Investigation of iron-sulfur protein maturation in eukaryotes. *Methods Mol Biol* **372**, 325-342, doi:10.1007/978-1-59745-365-3_24 (2007).

4 Stehling, O., Sheftel, A. D. & Lill, R. Chapter 12 Controlled expression of iron-sulfur cluster assembly components for respiratory chain complexes in mammalian cells. *Methods Enzymol* **456**, 209-231, doi:10.1016/S0076-6879(08)04412-1 (2009).

5 Schindelin, J. *et al.* Fiji: an open-source platform for biological-image analysis. *Nat Methods* **9**, 676-682, doi:10.1038/nmeth.2019 (2012).

6 Zucca, F. A. *et al.* Neuromelanin organelles are specialized autolysosomes that accumulate undegraded proteins and lipids in aging human brain and are likely involved in Parkinson's disease. *NPJ Parkinsons Dis* **4**, 17, doi:10.1038/s41531-018-0050-8 (2018).

7 Hughes, C. S. *et al.* Single-pot, solid-phase-enhanced sample preparation for proteomics experiments. *Nat Protoc* **14**, 68-85, doi:10.1038/s41596-018-0082-x (2019).

8 Demichev, V., Messner, C. B., Vernardis, S. I., Lilley, K. S. & Ralser, M. DIA-NN: neural networks and interference correction enable deep proteome coverage in high throughput. *Nat Methods* **17**, 41-44, doi:10.1038/s41592-019-0638-x (2020).

9 Tyanova, S. & Cox, J. Perseus: A Bioinformatics Platform for Integrative Analysis of Proteomics Data in Cancer Research. *Methods in molecular biology (Clifton, N.J.)* **1711**, 133-148, doi:10.1007/978-1-4939-7493-1_7 (2018).

10 Tusher, V. G., Tibshirani, R. & Chu, G. Significance analysis of microarrays applied to the ionizing radiation response. *Proceedings of the National Academy of Sciences* **98**, 5116-5121, doi:10.1073/pnas.091062498 (2001).

11 Huang da, W., Sherman, B. T. & Lempicki, R. A. Systematic and integrative analysis of large gene lists using DAVID bioinformatics resources. *Nat Protoc* **4**, 44-57, doi:10.1038/nprot.2008.211 (2009).

12 Sherman, B. T. *et al.* DAVID: a web server for functional enrichment analysis and functional annotation of gene lists (2021 update). *Nucleic Acids Res* **50**, W216-W221, doi:10.1093/nar/gkac194 (2022).
